# The potential impact of climate change on Australia's soil organic carbon resources

**DOI:** 10.1186/1750-0680-1-14

**Published:** 2006-12-06

**Authors:** Peter R Grace, Wilfred M Post, Kevin Hennessy

**Affiliations:** 1 School of Natural Resource Sciences and Institute for Sustainable Resources, Queensland University of Technology, GPO Box 2434, Brisbane, QLD 4001, Australia; 2 Oak Ridge National Laboratory, Oak Ridge, TN 37831, USA; 3 CSIRO Marine and Atmospheric Research, Private Bag No. 1, Aspendale, VIC 3195, Australia

## Abstract

**Background:**

Soil organic carbon (SOC) represents a significant pool of carbon within the biosphere. Climatic shifts in temperature and precipitation have a major influence on the decomposition and amount of SOC stored within an ecosystem and that released into the atmosphere. We have linked net primary production (NPP) algorithms, which include the impact of enhanced atmospheric CO_2 _on plant growth, to the SOCRATES terrestrial carbon model to estimate changes in SOC for the Australia continent between the years 1990 and 2100 in response to climate changes generated by the CSIRO Mark 2 Global Circulation Model (GCM).

**Results:**

We estimate organic carbon storage in the topsoil (0–10 cm) of the Australian continent in 1990 to be 8.1 Gt. This equates to 19 and 34 Gt in the top 30 and 100 cm of soil, respectively. By the year 2100, under a low emissions scenario, topsoil organic carbon stores of the continent will have increased by 0.6% (49 Mt C). Under a high emissions scenario, the Australian continent becomes a source of CO_2 _with a net reduction of 6.4% (518 Mt) in topsoil carbon, when compared to no climate change. This is partially offset by the predicted increase in NPP of 20.3%

**Conclusion:**

Climate change impacts must be studied holistically, requiring integration of climate, plant, ecosystem and soil sciences. The SOCRATES terrestrial carbon cycling model provides realistic estimates of changes in SOC storage in response to climate change over the next century, and confirms the need for greater consideration of soils in assessing the full impact of climate change and the development of quantifiable mitigation strategies.

## Background

Globally, the amount of carbon stored in soils is over three times that found in the atmosphere [1]. Soil organic carbon (SOC) is essential for maintaining fertility, water retention, and plant production in terrestrial ecosystems [2]. The amount of SOC stored within an ecosystem, is dependent on the quantity and quality of organic matter returned to the soil matrix, the soils ability to retain organic carbon (a function of texture and cation exchange capacity), and biotic influences of both temperature and precipitation [3]. The global decline in SOC as a result of deforestation, shifting cultivation and arable cropping have made significant contributions to increased levels of atmospheric CO_2 _[4].

Eventhough Australia is the world's smallest continent, its land mass is equivalent to 80% of continental Europe, and therefore its soils provides a significant source of CO_2 _if released to the atmosphere. A widespread reduction in SOC with a concomitant decline in soil structure has been the catalyst for soil crusting and compaction [5]. Infiltration rates are decreasing with subsequent losses of soil through both wind and water erosion thus increasing the potential for desertification. It has been estimated that up to 39% of organic carbon in cultivated surface soils of Australia has been lost between 1860 and 1990 [6]. Many of the soils are very old and have developed on heavily weathered parent surfaces [7]. The strong link between phosphorus availability and plant production [8] also means nutrient deficiencies are then compounded by a subsequent reduction in plant production, meaning less organic material returning to the soil matrix through decomposition.

The abiotic influences on SOC dynamics, such as moisture, temperature, aeration and the composition of plant residues are reasonably well understood. Because SOC storage is soil and environment dependent we consider an examination of the full potential of the effects of climate on SOC cycling in terrestrial ecosystems can only be assessed in the context of whole system simulation models. These allow the integration of the many basic empiricisms describing the processes and properties in carbon turnover and allow feedbacks of primary production and climate to be coupled.

It is within this context we use a relatively simple model of soil carbon dynamics, SOCRATES [2,3] to evaluate the impacts of future climate change on SOC stores of Australia, for each of the distinct biogeographical regions of Australia. SOCRATES encapsulates our current knowledge of SOC dynamics and litter decomposition in terrestrial ecosystems having been successfully tested against a global dataset [3] to estimate changes in SOC in response to both biotic and abiotic influences.

## Results and discussion

In the steady state analysis, simulated annual net primary production (NPP) for Australia averaged 8.1 t dry matter ha^-1 ^and ranged from 2.9 t ha^-1 ^in the Simpson Strzelecki Dune region to 18.3 t ha^-1 ^in the Top End Coastal biogeographic region of northern Australia (see Table 1). These are in general agreement with earlier predictions when using the Miami model to estimate NPP across Australia [9]. Our simulated estimate of the 1990 baseline value for SOC in the topsoil (10 cm) is 8.1 Gt. This value is 31% higher than our simplified literature analysis of SOC based on previously published data [10]. In our derivation we only used a mean literature value of SOC for each soil type, independent of its geographic location and nature and the vegetation present.

**Table 1:** The effect of climate change on SOC (0–10 cm) and net primary production (NPP) between 1990 and 2100 for the major biogeographic regions of Australia.

IBRA^*a *^Region	IBRA Code	SOC Lit.^*b *^1990 (Mt C)	SOC Sim. (A) 1990 (Mt C)	SOC B-A (%)^*c*^	SOC C-A (%)^*c*^	NPP A (g m^-2^)	NPP B-A (%)^*c*^	NPP C-A (%)^*c*^
Australian Alps	AA	27.4	38.1	0.4	-3.7	1135	5.6	11.9
Avon Wheatbelt	AW	97.9	117.0	0.4	-6.3	633	6.3	21.1
Ben Lomond	BEN^*d*^	52.7	86.2	0	-4.5	1023	6.4	17.8
Brigalow Belt North	BBN	229.7	203.7	0.9	-6.5	1039	13.0	45.2
Brigalow Belt South	BBS	408.8	571.4	0.8	-4.0	990	9.8	31.0
Broken Hill Complex	BHC	59.3	46.1	-0.4	-11.3	438	10.3	35.1
Burt Plain	BRT	28.0	37.9	0	-9.4	353	5.9	6.3
Central Arnhem	CA	33.6	39.7	0.8	-10.9	1177	8.9	18.1
Carnarvon	CAR	35.9	43.8	0.9	-6.7	377	12.8	43.6
Central Highlands	CH^*d*^	151.9	171.4	-0.5	-9.6	1585	10.9	38.9
Channel Country	CHC	119.2	149.6	0.6	-7.2	429	8.5	20.8
Central Kimberly	CK	40.0	40.0	2.2	-3.6	590	9.2	19.4
Central Mackay Coast	CMC	33.6	23.5	1.4	-6.4	1286	11.0	33.9
Coolgardie	COO	122.3	129.2	0.2	-6.4	491	6.3	13.0
Cobar Peneplain	CP	66.9	76.0	0	-8.9	540	12.7	47.8
Central Ranges	CR	37.9	54.6	0.7	-6.1	421	4.8	-0.7
Cape York Peninsular	CYP	150.1	174.6	2.7	-4.1	1808	9.8	23.0
D'Entercasteaux	DE	In BEN						
Daly Basin	DAB^*d*^	47.1	71.6	2.3	-5.8	1427	6.4	0.5
Desert Uplands	DEU	80.5	92.8	0.6	-8.9	918	9.5	28.6
Damperland	DL	46.6	53.0	2.0	-4.8	697	8.5	15.0
Darling Riverine Plain	DRP	137.2	225.2	0.3	-6.5	958	11.8	43.0
Einasleigh Uplands	EIU	124.9	206.6	1.3	-5.9	1372	8.1	17.0
Esperance Plains	ESP	46.0	46.1	-0.1	-6.9	542	7.2	19.9
Eyre and York Blocks	EYB	78.9	115.3	0	-6.9	797	6.8	17.5
Finke	FIN	29.3	48.4	-0.2	-8.1	373	2.8	-9.5
Flinders/Olary Ranges	FOR	30.2	54.6	-0.2	-7.4	455	2.2	-10.6
Freycinet	FRE	In BEN						
Gascoyne	GAS	70.7	94.7	1.0	-5.6	419	8.6	20.0
Gawler	GAW	31.4	49.0	0.1	-7.0	514	8.3	23.9
Gibson Desert	GD	60.7	98.7	0.5	-4.6	331	6.8	14.1
Gulf Fall and Uplands	GFU	69.6	90.5	0.7	-10.1	768	7.9	13.8
Geraldton Sandplains	GS	44.8	24.8	1.3	-4.3	400	9.6	25.3
Great Sandy Desert	GSD	153.9	216.6	1.1	-5.5	437	7.1	10.2
Gulf Coastal	GUC	18.1	25.2	1.0	-9.2	959	8.7	17.4
Great Victoria Desert	GVD	192.8	306.6	0.2	-6.2	383	5.0	3.0
Gulf Plains	GUP	165.0	192.5	1.9	-4.5	951	8.9	18.6
Hampton	HAM	4.8	10.6	-0.4	-7.4	459	2.3	-7.7
Jarrah Forest	JF	59.9	71.0	-0.1	-6.8	674	6.5	16.6
Lofty Block	LB	37.1	40.0	-0.6	-9.2	674	5.5	10.4
Little Sandy Desert	LSD	42.8	54.7	1.1	-5.5	361	9.4	23.3
MacDonnell Ranges	MAC	14.4	20.7	0	-8.4	373	3.4	-8.3
Mallee	MAL	103.8	96.3	0	-6.8	528	7.2	18.9
Murray-Darling Depr.	MDD	256.7	269.3	-0.3	-9.0	595	8.4	26.3
Mitchell Grass Downs	MGD	199.6	239.5	0.8	-8.1	621	8.6	18.6
Mount Isa Inlier	MII	43.2	36.2	1.8	-4.2	482	9.4	22.8
Mulga Lands	ML	159.2	250.9	0.5	-7.3	669	11.2	37.3
Murchinson	MUR	156.8	185.6	0.7	-6.0	423	8.1	18.6
Nandawar	NAN	49.7	74.6	0.6	-3.2	1075	7.7	22.3
Naracoorte Coastal Pln.	NCP	67.6	104.6	-0.8	-8.7	1130	4.8	9.8
New England Tableland	NET^*d*^	193.4	234.9	1.1	-4.5	1729	7.5	18.5
North Kimberly	NK	45.2	78.1	2.3	-5.9	1149	10.1	23.2
NSW North Coast	NNC	In NET						
NSW SW Slopes	NSS	109.6	205.3	0.1	-6.0	945	10.2	36.7
Nullabor	NUL	101.4	157.9	0.2	-5.9	404	4.7	3.7
Ord-Victoria Plains	OVP	81.4	98.5	1.5	-6.9	775	8.1	13.4
Pilbara	PIL	69.9	97.3	1.6	-5.0	538	7.1	8.8
Pine Creek-Arnhem	PCA	in DAB						
Riverina	RIV	94.2	135.7	-0.1	-8.2	713	13.2	51.3
South East Corner	SEC	64.3	103.7	-0.1	-4.4	1050	5.5	14.9
South East Queensland	SEQ	143.0	150.7	1.2	-5.9	1596	9.4	26.8
Simp.Strzelecki Dune	SSD	108.4	147.4	0.6	-4.5	293	5.5	6.1
SE Coastal Plain	SCP	36.7	58.7	-0.1	-5.8	1099	5.9	14.6
SE Highlands	SHE	178.9	243.7	0.1	-5.4	1015	8.2	26.4
Stony Plains	STP	70.8	94.3	0.1	-7.8	305	5.6	5.5
Sturt Plateau	STU	51.9	70.7	0.6	-10.7	713	7.6	11.0
Swan Coastal Plain	SWA	19.7	30.0	0.5	-7.1	1315	8.4	23.5
Sydney Basin	SB	85.8	122.2	0.1	-4.6	1072	6.7	19.5
Tanami	TAN	123.5	201.7	0.7	-8.7	578	5.8	2.3
Tasmanian Midlands	TM	in BEN						
Top End Coastal	TEC	62.5	76.6	2.5	-6.5	1827	9.1	16.4
Victorian Bonaparte	VB	66.4	79.0	2.9	-3.5	1427	8.0	10.6
Vic Midlands	VM^*d*^	92.3	176.6	-0.5	-8.2	1062	6.4	17.5
Vic Volcanic Plain	VVP	in VM						
Warren	WAR	13.6	26.0	-0.2	-6.9	987	6.9	19.1
West and South West	WSW	in CH						
Woolnorth	WOO	in CH						
Wet Tropics	WT	24.1	29.4	1.2	-9.8	1542	12.1	37.5
Yalgoo	YAL	14.1	20.9	0.8	-5.6	415	8.1	21.9
Australian continent^*e*^		6169.0	8107.0	0.6	-6.4	811	8.2	20.3

^*a*^Interim Biogeographic Regionalization for Australia

^*b*^based on literature values of Spain et al. (1983)

^*c*^comparison of scenarios based on difference and expressed as a percentage where Scenario A, B and C are no, low and high global climate sensitivity to increased greenhouse gas concentrations, respectively

^*d*^composite value

^*e*^excludes Furneaux chain of islands

Organic carbon concentration characteristically declines down the soil profile. The topsoil value is approximately 43 and 24% of the total organic carbon stored in the top 30 and 100 cm, respectively [11]. Our simulated baseline estimate (Scenario A) of SOC storage for the continent in the top 30 cm is 18.8 Gt and 34.2 Gt in the top 100 cm. The latter value lies within the range of published estimates of 27 and 50 Gt [12,13].

Our estimates are based on combining a broad classification of Australian soils with summary soil survey data. The immense size of the Australian continent and the large amount of variability in SOC characteristically found across a landscape also places limits on the utility of the current store of soil survey information. The fact that our simulated baseline value for SOC lies in the range of literature values, leads us to believe our baseline estimate is entirely plausible.

In our low sensitivity to climate change simulation, Scenario B, the mean annual temperature of the continent is predicted to rise by 1.0°C by the year 2100 with no change in the mean annual precipitation. However, the spatial distribution of precipitation was slightly different than in our high sensitivity to climate change simulation, Scenario A. The CSIRO Mark 2 GCM predicted that the largest increase in annual precipitation (28 mm) for the continent would occur in the Riverina biogeographic region. Understandably, the largest predicted increase in annual NPP was also found in the same region (13.2% of the 1990 value).

Our simulations suggest little change in SOC storage when comparing the low sensitivity to climate change (Scenario B) with no climate change (in Scenario A). There is an average increase in topsoil organic carbon of only 0.6% (49 Mt) compared to the no climate change scenario for the continent as a whole by the year 2100, with an average topsoil organic carbon concentration of 10.6 t ha^-1^. This is associated with an 8.2 % increase in annual NPP (compared to the 1990 value), equivalent to 204 Mt C by 2100.

A slight decline in SOC is predicted in 15 of the biogeographic regions over the next century under the low sensitivity to climate change scenario (see Table 1). Some of the most significant reductions are geographically clustered in south-eastern Australia, specifically the Broken Hill Complex, Lofty Block, Murray Darling Depression, Naracoorte Coastal Plain, Riverina, Victorian Bonaparte, and Victorian Volcanic Plain biogeographic regions. This area comprises the Wimmera district in western Victoria extending west into South Australia and south-west New South Wales, and is a major contributor to cereal production in southern Australia. For Lofty Block, Naracoorte Coastal Plain, Victorian Bonaparte and the Victorian Volcanic Plain, the decline is driven by temperature increases that are greater than the continental average combined with less then average increases in NPP by the year 2100.

Under the low sensitivity to climate change scenario, the largest increases in topsoil organic carbon over the next century (2.0–2.9%) are predicted for the biogeographic regions located in the tropics, i.e. the Kimberley and Arnhem districts in the north of Western Australia and the Northern Territory respectively, and Cape York Peninsula in northern Queensland. Specifically, the Central Kimberley, Cape York Penisular, Daly Basin, Dampierland, North Kimberley, Pine Creek Arnhem, Top End Coastal, and Victorian Bonaparte biogeographic regions. In these regions, annual NPP by the year 2100 will average 13.9 t ha^-1 ^(compared to the continental average of 8.8 t ha^-1^) with average annual mean temperatures exceeding 27°C.

In Scenario C, the mean annual temperature of the continent increases 5.3°C by the end of the year 2100 with no change in the mean annual precipitation. The rainfall distribution was the same as that in Scenario B, but the magnitude of the changes in precipitation in the various regions was more extreme than predicted in Scenario B. In Scenario B, the changes in precipitation within regions ranged from -11 mm to + 28 mm. In Scenario C, the magnitude ranged from -54 mm to + 144 mm. In the high sensitivity to climate change scenario, there is a predicted increase in annual NPP for the continent of 1.6 t ha^-1 ^by the year 2100 compared to the no climate change scenario. This is 11.2% higher than the low sensitivity to climate change scenario and 20.3% higher than if there was no climate change. Increases in annual NPP in excess of 40% of the 1990 estimates are predicted for the Brigalow Belt North, Carnarvon, Central Highlands, Cobar Peneplain, Darling Riverine Plain, and Riverina biogeographic regions (see Table 1), with the latter, a major cereal production region, exceeding 50% by the year 2100.

Under the high sensitivity to climate change scenario, topsoil organic carbon stocks for the continent are estimated to be 7.6 Gt C in 2100, with an average concentration of 9.9 t ha^-1^. Compared to the steady state value in 1990, this is an overall reduction in topsoil organic carbon for the continent of 6.4%, or 519 Mt. This reduction is partially offset by an increase in continental NPP of 505 Mt C.

In 4 biogeographic regions, SOC is predicted to decline by at least 10% compared to the 1990 baseline value. Three of these regions (Central Arnhem, Gulf Fall & Uplands, Sturt Plateau) are located in the northern tropics where annual rainfall will potentially decline by an average of 57 mm. The predicted increase in annual NPP for these regions (14.3%) is well below the continental average (20.3%) and the mean annual temperature at these sites is 1.5°C higher than the continental average for Scenario C. It is interesting to note that these same 3 regions had actually accumulated slightly higher than average amounts of SOC in Scenario B but in Scenario C were now the greatest sources of terrestrial carbon emissions on the continent.

The biogeographic regions that showed the least change in SOC (Brigalow Belt South, Central Kimberley, Nandawar, Victorian Bonaparte) were not geographically clustered as was the case for similar performing regions in Scenario B, but the average increase in temperature was only 4.3°C, a degree lower than the average temperature predicted for the continent in Scenario C.

## Conclusion

Climate change effects on the terrestrial carbon cycle are principally driven by the response of vegetation to these changes. Decomposition of organic material is also influenced by the same temperature and precipitation inputs which drive vegetative growth. We have attempted to predict the impact of these environmental variables on the cycling of SOC in terrestrial ecosystems of Australia through the 21st century in response to climate change. Using a relatively simple simulation model of SOC dynamics, we consider Australia's SOC resources to be a sink for carbon over the next century if there is no or little global climate sensitivity to greenhouse emissions.

If we consider a worst case scenario, a high global climate sensitivity to emissions, the continent's soils will be a source of emissions. In the latter scenario, SOC in the top 10 cm of the Australian continent will be depleted by 0.5 Gt by the year 2100 when compared to no climate change, however this may be partially offset by an increase in total NPP of a similar magnitude. Our observations are consistent with simulations in other regions of the globe [14,15], in that predicted changes in temperature and moisture over the next century will significantly increase the rate of decomposition in soils and reduce organic carbon stocks. The loss of carbon will be partially slowed by increases in carbon inputs due to increases in NPP.

Our study also strongly supports the need for a more thorough systems approach to assessing the impact of climate change on the many interlinked components of the global carbon cycle [16], with an increased emphasis on soils [17]. A full carbon accounting approach is of particular relevance when developing quantifiable mitigation strategies for credit, as concurrent impacts on the decomposition of carbon from soils will potentially discount aboveground carbon assets.

There are a number of limitations to our approach which must be taken into account for improving future studies. Total NPP, especially the allocation of biomass belowground, is difficult to quantify, especially soluble carbon inputs as root exudations. The influence of nitrogen and phosphorus also needs to be factored into NPP estimates, however this is model dependent. In this study we have used a minimum dataset approach, to demonstrate the utility of simple models in examining the impact of climate change on terrestrial carbon cycling. In our simulations, we have also assumed that land use does not change in response to climate change Projecting future land-use change is another component which could have as great an impact on continental SOC storage climate and CO_2 _changes. For increased accuracy, successional change algorithms should also be included into our models as species composition has a major bearing on nutrient turnover. Spatial variability is also a major problem and soil survey data available for Australia is problematic considering the size and geographic diversity of the country. Surveying is an expensive exercise but with the on-going advances in geostatistical and interpolation theory, and remote sensing, the accuracy in soil maps may be greatly improved at the landscape and even finer scales.

Simulation models of SOC cycling are improving as modellers work more closely with the experimental scientist in developing models and techniques that allow them to use easily measurable fractions or surrogates based on pedotransfer functions. The wealth of knowledge which has gone into the development of simulation models such as SOCRATES, RothC [18], CENTURY [19] and Introductory Carbon Balance Model (ICBM) [20], and the fact that they do not require detailed analyses to perform accurate simulations, provides a promising avenue for policy makers to predict the impacts of climate change on the cycling of SOC in terrestrial ecosystems.

While the current models of vegetation-soil-atmosphere interactions have limitations we are confident they provide reasonable estimates of potential response to climate change. More importantly, our exercise clearly shows that climate change impacts must be studied holistically, requiring continued integration of climate, plant, ecosystem and soil sciences. In this way, processes can be examined in more detail and strategies developed which will actually be used for adaptation to, or mitigation of, climate change and its effects. Laboratory, field and atmospheric observations must be integrated through a systems approach that simulation modelling can provide.

## Methods

**Figure 1:**
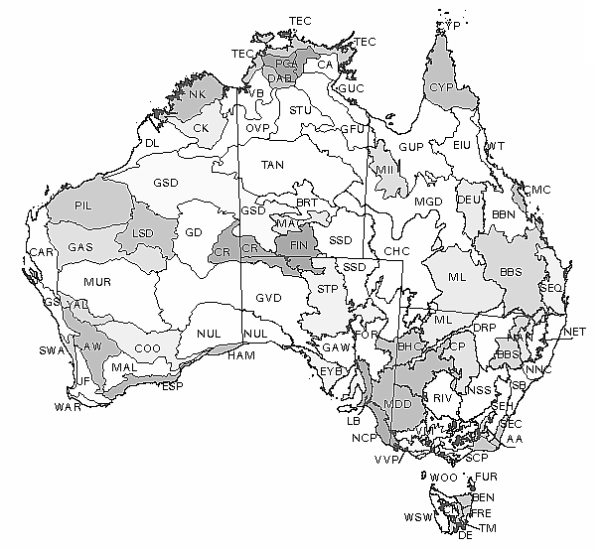
Geographic location of biogeographic regions of Australia as classified by the Interim Biogeographic Regionalisation for Australia (IBRA).

The Interim Biogeographic Regionalization for Australia (IBRA) [21] was used to subdivide the Australian continent into 80 distinctive ecosystems. The geographic location of each region is shown in Figure 1. We overlaid a 2.5° × 2.5° climate grid and allocated a soil classification and texture to each grid cell by overlaying an eighteen class soil map of Australia [22]. Soil texture data was converted to a CEC value (mmol kg^-1^) using a linear relationship [23]. Bulk density was assumed to be 1.3 g cm^-3 ^for all soils with mean annual temperature and precipitation for each cell extracted from historical climate records.

A simulation methodology [24] was then used to generate a baseline SOC map of Australia. All grid cells were assigned an initial value of 0.1% SOC prior to generating the steady-state values in 1990. Potential changes in SOC in each grid cell and biogeographic region between 1990 and 2100 were then simulated under a range of climate scenarios. This assessment is thus based on changes in net primary production (NPP) (gross photosynthetic carbon fixation less plant respiration) and SOC dynamics in response to predicted temperature and precipitation as provided by the CSIRO Mark 2 GCM.

We compared two possible climate change scenarios (B and C) generated by the GCM with a baseline scenario (A) which was used to estimate the steady state value for SOC in 1990. Scenario A assumes no climate change over the next century. Annual mean temperatures and precipitation for each grid cell remained fixed at long-term average values applicable in 1990. For the NPP estimations in scenario A we assumed atmospheric CO_2 _concentration remained constant at the 1990 concentration of 350 ppm. Scenario B represents low global climate sensitivity to increased greenhouse gas concentrations. For the purposes of representing a realistic vegetation response we assumed that atmospheric CO_2 _concentrations gradually increased to 520 ppm over the next century. Scenario C represents high global climate sensitivity to increased greenhouse gas concentrations. In this scenario we assumed that atmospheric CO_2 _concentrations increased to 1080 ppm by the end of the next century. Note that there is a distinction between local or landscape level changes in climate and global climate sensitivity, with the former being scaled by the latter.

The scenarios take into account the Intergovernmental Panel on Climate Change (IPCC) range of greenhouse gas emission scenarios (IS92a-f), and the IPCC range of climate sensitivity (a global mean warming of 1.5°C to 4.5°C for an equivalent doubling of pre-industrial CO_2 _concentrations). Scenarios B and C represent the extreme differences i.e.

(i) low emissions (IS92c) and low climate sensitivity (1.5°C) and

(ii) high emissions (IS92e) and high climate sensitivity (4.5°C) respectively.

The radiative effect of stratospheric ozone depletion was included (which offsets greenhouse warming) when generating these temperature and precipitation scenarios, but the radiative effect of sulfate aerosols was excluded (which also offsets greenhouse warming) because it is difficult to predict in the southern hemisphere. The climate scenarios do not take into account the CO_2 _fertilisation of the biosphere (resulting in increased uptake of CO_2 _by plants, thereby reducing the rate of increase of atmospheric CO_2_, and hence global warming), but we did explicitly include CO_2 _fertilization in our net primary productivity calculations. This approach assumes that the pattern of regional climate response at any time in the future will be in proportion to the pattern of response for an equivalent doubling of the concentration of CO_2 _as simulated in climate models. It does not allow for possible changes in the El Nino Southern Oscillation behaviour, an important climatic feature not simulated well in climate models. Possible changes in ocean circulation are also not accounted for, and provide a major uncertainty about future climate change. In our case, the seasonal changes in temperature and precipitation provided by the GCM were averaged for each year to provide annual values for input into the SOCRATES model.

SOCRATES, or **S**oil **O**rganic **C**arbon **R**eserves **A**nd **T**ransformations in **E**co**S**ystems, is a simulation model originally designed to estimate changes in SOC (0–10 cm) in agricultural systems as influenced by management and climate [25]. The versatility of SOCRATES in predicting soil carbon change across a wide range of terrestrial ecosystems and management interventions has been demonstrated [2,3]. The accuracy of SOCRATES was also reflected in an extensive model comparison [26]. They found it to be superior to both the CENTURY and RothC-26.3 models (amongst others) in predicting long-term changes in SOC in production systems of the Canadian prairies. For the purposes of this paper we have replaced the original plant production component of SOCRATES with a modification of the Miami model of net primary productivity (NPP) [27] as previously described [24]. This modification reflects any changes in NPP (g m^-2 ^yr^-1^) in response to changes in atmospheric CO_2 _concentration and is represented by the following equation.

NPP = min(NPP_T_,NPP_P_)(1+β (p-p_0_)/p_0_)     (1)

where "min" is a function which selects the minimum value from the two NPP calculations NPP_T _and NPP_P_, which are based on mean annual temperature T (°C) and average annual precipitation P (mm), respectively. Explicitly,

NPP_T _= 3000/(1+e^1.315-0.119T^)     (2)

NPP_P _= 3000(1-e^-0.000664P^)     (3)

The Miami model was originally derived from 52 locations around the globe and whilst we recognise the shortcomings of its simplicity, it has an advantage over site-specific regressions in that it is valid over a range of climates far exceeding those normally experienced at a single location in Australia [9]. The model is also limited to estimating the primary productivity of what may be considered stable or climax vegetation (the vast majority of the Australian continent), and is less reliable for arable cropping sites.

The CO_2 _response coefficient β [28] is based on leaf photosynthetic response of C_3 _plants. Its direct application to an ecosystem level community has not been verified, therefore we have followed a global simulation example [24] in which β is reduced to 60% of the calculated value by the use of a scale translation factor. This conversion is based on experimental studies on biomass response and photosynthetic assimilation to elevated CO_2 _[29]. The state variables p and p_0 _represent atmospheric and reference CO_2 _concentrations respectively.

Carbon inputs for any terrestrial ecosystem can be derived by assuming dry matter contains 40% carbon and firstly partitioning NPP into leaf, branch, stem and roots. As we are simulating SOC dynamics in the top 10 cm, annual root production in this layer was allocated as outlined in Table 2[30]. The carbon density of each plant component at steady state (B) is estimated using equation 4.

**Table 2:** Parameters used to simulate litter production and soil organic matter dynamics for biomes of Australia.

	Partition Coefficient				Life span (yrs)				DPM/RPM^*a*^	Root dist.^*b*^
	
Biome	Leaf	Branch	Stem	Root	Leaf	Branch	Stem	Root		
Tropical forest	0.3	0.2	0.3	0.2	1	10	50	10	0.3	0.33
Savanna	0.6	0.1	0.1	0.2	1	5	30	12	0.3	0.25
Temperate forest	0.3	0.2	0.3	0.2	2	10	60	10	0.2	0.25
Grassland	0.6	0.0	0.0	0.4	1	-	-	1	0.4	0.35
Arable	0.8	0.0	0.0	0.2	1	-	-	1	0.59	0.33
Desert	0.55	0.05	0.05	0.35	1	10	40	1	0.33	0.22
Shrubland	0.5	0.1	0.1	0.3	1	10	50	2	0.4	0.31

^*a*^Ratio of decomposable to resistant plant material.

^*b*^Proportion of roots in top 10 cm of soil.

B = NPPpY     (4)

where NPP is annual NPP (from equation 1), p is the partitioning coefficient for each of the plant components and Y is the average life span (in years), for the component. We classified each of the 80 biogeographic regions into one of seven biomes and used partitioning constants and average life spans as outlined in Table 2[28]. The annual litter carbon input (L) for each plant component is then estimated by equation 5.

L = (1/Y)B     (5)

The SOCRATES model is based on four major organic carbon pools, two soil and two litter. All plant material can be divided into decomposable and resistant components [31]. The decomposable plant material (DPM) is readily degraded by microbes and is related to the more succulent parts of the plant. It mainly consists of sugars and carbohydrate. The resistant plant material (RPM) is associated with the woody structure of the plant and usually consists of cellulose and lignin. The respective DPM/RPM ratios for the litter produced in each biome [32] are outlined in Table 2. The soil pools consist of microbial biomass (BIO) and stable organic matter or humus (HUM). The microbial fraction is further subdivided into a transient unprotected fraction, which is involved in the initial stages of crop residue decomposition and a protected microbial fraction which is actively involved in the decomposition of native humus and microbial metabolites [33].

The generic description of decomposition in the model produces microbial material, humus and CO_2 _in proportions which are dependent on soil texture, or more specifically the cation exchange capacity of a soil. These proportions and the specific decay rates for each pool of the model were calibrated using ^14^C data [33]. The first order decay rates currently used in the model are 0.84 w^-1 ^for decomposable plant material (i.e. 84% of the material will degrade in one week at 25°C at optimum moisture conditions), 0.07 w^-1^, 0.95 d^-1^, 0.055 w^-1^ and 0.0009 w^-1 ^for resistant plant material, unprotected and protected microbial biomass and stable organic matter pools respectively.

The decay rate for the resistant plant fraction in SOCRATES is significantly faster than those specified in the CENTURY and RothC-26.3 models. By definition we consider this material to be recognisable light fraction which is capable of being removed prior to a SOC analysis being performed. Decay rates are modified using multiplicative scalars of annual mean temperature and average precipitation (as a surrogate for soil moisture). The effect of temperature on decomposition is based on a Q_10 _relationship of 2.0 [3]. The moisture scalar ranges from 0.25 to 0.45 as annual average precipitation increases to 1400 mm.

Steady state values of SOC for the year 1990 were estimated after running the modified SOCRATES model to equilibrium. In this case, the model was initialized with a minimal SOC content, with 3% of the initial SOC considered to be protected microbial biomass and the remaining 97% stable humus. Once steady state was reached, the final values for both these components were used to re-initialize the model for the post-1990 scenario analysis.

## Competing interests

The author(s) declare that they have no competing interests.

## Authors' contributions

PG performed the simulations, data synthesis and interpretation and wrote the manuscript, WMP provided assistance in model development and methodology, KH provided regional GCM outputs. All authors read and approved the final manuscript.
